# Cancer‐associated fibroblasts nurture LGR5 marked liver tumor‐initiating cells and promote their tumor formation, growth, and metastasis

**DOI:** 10.1002/cam4.6408

**Published:** 2023-08-14

**Authors:** Mingna Zhang, Yiqiao Fang, Xia Fu, Jiaye Liu, Yang Liu, Zhounan Zhu, Yinyun Ni, Menglin Yao, Qiuwei Pan, Wanlu Cao, Zhihui Li, Chunyan Dong

**Affiliations:** ^1^ Department of Oncology Postgraduate Training Base of Jinzhou Medical University, Shanghai East Hospital Shanghai China; ^2^ Department of Thyroid and Parathyroid Surgery, West China Hospital Sichuan University Chengdu Sichuan China; ^3^ Laboratory of Thyroid and Parathyroid Diseases, Frontiers Science Center for Disease‐Related Molecular Network, West China Hospital Sichuan University Chengdu Sichuan China; ^4^ Department of Outpatients, West China Hospital Sichuan University Chengdu Sichuan China; ^5^ Department of Obsterics and Gynecology, Second Affiliated Hospital Chongqing Medical University Chongqing China; ^6^ Department of Oncology Shanghai East Hospital, Tongji University School of Medicine, Tongji University Shanghai People's Republic of China; ^7^ Department of Respiratory and Critical Care Medicine, National Clinic al Research Center for Geriatrics, Center of Precision Medicine, Precision Medicine Key Laboratory of Sichuan Province, Frontiers Science Center for Disease‐related Molecular Network, West China Hospital, West China School of Medicine Sichuan University Chengdu Sichuan China; ^8^ Department of Gastroenterology and Hepatology Erasmus Medical Center Rotterdam the Netherlands; ^9^ Department of Oncology, Shanghai Key Laboratory of Chemical Assessment and Sustainability, School of Chemical Science and Engineering East Hospital Affiliated to Tongji University, Tongji University School of Medicine, Tongji University Shanghai People's Republic of China

**Keywords:** cancer‐associated fibroblasts, diphtheria toxin receptor, leucine‐rich repeat‐containing G‐protein coupled receptor 5, liver cancer, tumor‐initiating cells

## Abstract

**Background & Aims:**

In liver cancer, leucine‐rich repeat‐containing G‐protein coupled receptor 5 (LGR5) compartment represents an important tumor‐initiating cell (TIC) population and served as a potential therapeutic target. Cancer‐associated fibroblasts (CAFs) is a critical part of the tumor microenvironment, heavily influenced TIC function and fate. However, deeply investigations have been hindered by the lack of accurate preclinical models to investigate the interaction between CAFs and TIC. Organoids model have achieved major advancements as a precious research model for recapitulating the morphological aspects of organs, and thus also serving as a candidate model to investigate the mutual interaction between different cell types. Consequently, this study aimed to construct a three‐dimensional (3D) co‐culture organoid model of primary LGR5‐expressing tumor stem cells from primary murine liver tumors with CAFs to investigate the impact of CAFs on LGR5 marked TICs in liver cancer.

**Materials and Methods:**

First, both of the transgenic LGR5‐diphtheria toxin receptor (DTR)‐GFP knock‐in mice and transgenic Rosa26‐mT mice developed primary liver tumors by diethylnitrosamine (DEN) administration. Tumor organoids and CAFs were generated from those primary liver cancer separately. Second, LGR5‐expressing TICs organoid with CAFs were established ex vivo based on cell–cell contact or trans‐well co‐culture system, and the mutual influence between those two types of cells was further investigated. Subsequently, immunodeficient mouse‐based xenograft model was further adopted to evaluate the influence of CAFs to LGR5 tumor stem cell, tumor formation, and metastasis.

**Results:**

The co‐culture organoid model composed of murine liver tumor LGR5+ tumor‐initiating cells and CAFs in 3D co‐culture was successfully established, with the intention to investigate their mutual interaction. The existence of CAFs upon engrafting tumor organoids resulted in dramatic higher number of LGR5+ cells in the neoplasia when compared with engrafting tumor organoids alone. Furthermore, ex vivo culture of isolated LGR5+ cells from tumors of co‐engrafted mice formed significantly larger size of organoids than mono‐engrafted. Our results also indicated significantly larger size and number of formed organoids, when LGR5+ cells co‐cultured with CAF in both cell–cell contact and paracrine signaling in vitro*,* comparing to LGR5+ cells alone. Furthermore, we found that specific knockout of LGR5 expressing cells suppressed CAF‐mediated promotion of tumor formation, growth, and metastasis in the experimental mice model.

**Conclusions:**

Altogether, in a 3D co‐culture type of murine liver LGR5+ cells and cancer‐associated fibroblasts, we have demonstrated robust effects of CAFs in the promotion of LGR5 marked liver TICs. We also further revealed the influence of tumor microenvironment on stem cell‐related therapy, suggesting the possibility of combing CAF‐targeted and tumor stem cell targeted therapy in treating liver cancer.

## 1 INTRODUCTION

Liver cancer is one of the most common and deadly malignancies worldwide with limited treatment options available. There are over 1.2 million deaths yearly caused by liver cancer worldwide.^1^ Although systemic therapies for liver cancer, such as immune checkpoint inhibitors (ICIs), tyrosine kinase inhibitors (TKIs), and monoclonal antibodies, have made significant advancements in recent years. Heterogeneity within and between liver tumors greatly complicates disease progression and treatment response.^2^, ^3^, ^4^ Therefore, further research on liver cancer is required to provide novel and efficient therapies.

Tumor initiating cells (TICs), also known as cancer stem cells, are capable of both self‐renewal and lineage differentiation. These cells are thought to be responsible for tumor initiation, disease progression, and resistance to treatment.^5^, ^6^, ^7^ Recent studies have demonstrated that LGR5 expressing population is an important TICs population in liver cancer. The LGR5 compartment has since been further revealed as a potential therapeutic target.^8^, ^9^ Although LGR5 therapeutic targeting preclinical research already obtain fascinating progression,^10^ current clinical trials still lack impressive clinical benefit. Therefore, there is an urgent need for a more comprehensive mechanism research, especially the exploration of the reasons affecting the efficacy.

It is recognized that the tumor microenvironment significantly affects the role and course of tumor‐initiating cells.^11^ The term “Tumor microenvironment” refers to the cellular setting in which tumors or cancer stem cells resides. The setting is made up of surrounding blood vessels, immune cells, fibroblasts, and extracellular matrix.^12^ Among them, CAFs are the most abundant mesenchymal cells, which enable cancer cells to migrate from the primary tumor site into the bloodstream for systemic metastasis. Extensive fibrosis is brought on by fibroblast activation, proliferation, and accumulation in around 80% of liver tumors.^13^ A variety of mechanisms are reported to modulate the progression of HCC by cancer‐associated fibroblasts, which including direct effects on cancer cells through the secretion of soluble factors and exosomes, as well as indirect effects via other stromal cells and the remodeling of the extracellular matrix (ECM).^14^ Published data also revealed that LGR5 expressing intestinal tumor stem cell will be dramatically influenced by the intestinal CAF.^15^ However, the interaction between LGR5+ cells and CAFs in HCC has rarely been explored and we hypothesis that CAF‐related microenvironment will influence the behavior of LGR5 expressing tumor stem cell.

In the study of tumor microenvironment, neither 2D cell cultures nor in vivo xenografts or genetically modified animals are sufficient to mimic native human tumor immunology. The traditional method of simulating tumor cell‐to‐cell communication has involved co‐culturing 2D cultures with exogenous heterogeneous cells.^16^, ^17^ Furthermore, adherent monolayer cancer cells are incapable of replicating 3D morphological features when reconstructed from endogenous intra‐tumoral stroma. Several studies have also demonstrated the use of patient‐derived xenografts (PDXs) to create humanized immune tumor models in immunodeficient mice with human immune cells, but there are still difficulties with cost, time, throughput, and immune compatibility.^18^, ^19^ Therefore, a reliable experimental system is needed to represent patient‐specific tumor–immune interactions.

Organoids are 3D micro‐organs cultured in vitro from adult stem cells, and their spatial distribution pattern is similar to that of in vivo tissues. Therefore, organoids can simulate the 3D structure and function of real organs in vivo.^18^ Organoids have been proven to be powerful models for understanding human disorders and exploring disease treatments.^19^ Many different kinds of organoid models, such as those of the brain, retina, kidney, lung, stomach, and liver, have been developed effectively.^20^, ^21^, ^22^, ^23^, ^24^ Researchers have suggested that intestinal organoids derived from a single LGR5+ stem cell may form crypt‐villus structures with stratified epithelium, displaying major intestinal cell types. These intestinal organoids have been used to simulate tissue regeneration and cancer development.^25^ In recent years, tumor organoids in co‐culture have been used to study the mutual interaction between difference cell types. For instance the co‐culture system of tumor organoids and peripheral blood lymphocytes has been developed, T cells that are tumor‐reactive can be expanded from peripheral blood and evaluated for their ability to kill tumors.^20^, ^26^ Therefore, organoids are a useful in vitro model that matches some of the shapes and functions of the respective organs in vivo and also a candidate model to research the interaction between different cell types.

In conclusion, we intend to establish the 3D co‐culture organoid model to investigate whether tumor microenvironment‐related CAFs will affect the behavior and stemness of LGR5‐expressing tumor stem cell in liver cancer and the possible further influence to the LGR5 liver tumor stem cell related therapeutic targeting treatment.

## 2 MATERIALS AND METHODS

### 2.1 Model of a primordial liver tumor

Leucine‐rich repeat‐containing G‐protein coupled receptor 5 green fluorescent protein and CreERT fusion protein (LGR5‐GFP‐creERT) transgenic mice (kindly provided by Yujun Shi) cross‐breed with Rosa 26‐iDTR mice which are particularly capable of co‐expressing the diphtheria toxin receptor (DTR) and green fluorescent protein (GFP) within the LGR5 promoter. Hence, GFP expression identifies LGR5+ cells, and DT administration specifically depletes LGR5‐GFP+ cells. Diethylnitrosamine (DEN) was injected intraperitoneally (Sigma‐Aldrich, 100 mg/kg) every week for 6–17 weeks into cross‐bred mouse (3–4 weeks). DEN induces liver tumors in transgenic and wild‐type mice, resulting in basophilic foci, hyperplastic nodules, hepatocellular adenomas, and ultimately liver cancer. DEN‐injected mice were killed 3–16 months after the last injection, and livers and tumors were collected for further examination. The same protocol was used for inducing histologically verified liver tumors in Rosa26‐membrane tomato mice.

### 2.2 Organoid culturing of mouse liver tumors

The liver tumor organoids were derived from DEN‐treated LGR5‐GFP‐creERT/Rosa26‐iDTR mice and confirmed to be liver tumors by histology. Tumor tissue was placed in PBS solution and cut into 2 mm tissue pieces using ophthalmic scissors, which were placed in collagenase type XI (0.5 mg/mL, C9407; Sigma), dispase (0.2 mg/mL, 17,105,041), DMEM and 1% fetal bovine serum in the digestion solution, 37°C 30 min, digest the single cell suspension. The cells isolated from the precipitate were treated with DMEM/F12 (12634‐010; Invitrogen) washed to remove digestive enzymes, and cells were then incubated with Matrigel (356,231; BD Bioscience). The expansion medium was added slowly after waiting for Matrigel to solidify. Compared to mouse organoid basal medium (OBM), organoid expansion medium (OEM) (DMEM/F12 supplemented with 1% penicillin/streptomycin (15140122), FGF10 (100 ng/mL), nicotinamide (10 mmol/L, N0636; Sigma‐Aldrich), 1% Glutamax (BE‐17‐605E/U1), 10 mmol/L HEPES (be‐17‐737E), Sigma Aldrich), EGF (50 ng/mL, PeproTech, epidermal growth factor), N2 (1%, vol/vol, 17,502,001), R‐spondin 1 (10% vol/vol), and HGF (50 ng/mL, PeproTech, hepatocyte growth factor) is used for proliferation of organoids. Organisms were cultivated for 4 days in organoid initiation media containing Wnt3a, noggin (10% vol/vol, conditioned medium produced by the Noggin‐expression 293 T cell line), and Y‐27632 (10.5 mol/L, Sigma‐Aldrich), Every 2 days, the medium was updated.

In this study, we used precooled OBM to collect and rinse organoids, centrifuged, mixed with matrix glue, and then added fresh expansion median for reseeding. In accordance with the growth of the organoids, 1:6–1:10 of the organoids were passed each week. To allow long‐term preservation of the organoids, we mixed the passaged organoids with cryopreservation solutions according to standard procedures (dimethyl sulfoxide 10% added to 90% fetal bovine serum). After thawing and resuscitation, organoids need to be washed once with OBM, with organoid initiation medium and Matrigel (356,231; Corning BV) were reseeded.

### 2.3 Alamar Blue Assay

Alamar Blue (1:20 in DMEM, Invitrogen) was incubated with cancer‐associated fibroblasts and organoids for 2 h (37°C), and then, medium was collected to analyze metabolic activity of the cells. A fluorescence plate reader (CytoFluor Series 4000; Perseptive Biosystems) was used to determine absorbance at 530/25 nm and 590/35 nm. A blank control was Matrigel with medium only.

### 2.4 Isolation and culture of cancer‐associated fibroblasts from mouse liver tumor

Cancer‐associated fibroblasts were isolated from histologically confirmed liver tumors of Rosa26‐membrane tomato mice induced with DEN. An outgrowth isolation method was used to isolate cancer‐associated fibroblasts. We added collagenase type IV (0.5 mg/mL, C7657; Sigma Aldrich), dispase (0.2 mg/mL, 17,105,041), and 1% fetal bovine serum warmed in a 37°C water bath for 30–60 min to help digest the tissue near the tumor's periphery. Following that, samples were quenched in 10% fetal calf serum (FCS) RPMI 1640 medium after being filtered through a 70‐um screen. When the tumor fragments pellet was placed in a 35 mm Petri dish, fibroblast‐like cells developed and adhered to its surface. A minimum of three generations of passage were carried out to avoid the contamination of established cell cultures with cancer cells. Every 2 days, the medium was replaced, and the fibroblasts were sub‐cultured at 80% confluence, banked, and utilized for investigations in passages 4–8. To exclude contamination from other cell types, the fibroblasts were immunofluorescence stained with the fibroblast markers epithelial cell marker (Epcam, 1:1000, ab71916; Abcam), FAP (1:500, ab28244; Abcam), and negative staining for HCC cells (AFP, 1:50, SAB3500533; Sigma‐Aldrich), a‐SMA (1:1000, ab124964; Abcam).

### 2.5 Co‐culturing cancer‐associated fibroblasts and tumor organoids

Organoids were collected using cold OBM and split up into tiny fragments by blowing and washing 20 times through a pipettor, which were further digested with trypsin–EDTA (37°C, 2 min; Gibco). Further isolation of single living cells was achieved using fluorescence‐activated cell sorting (BD FACS Canto II, Flow Cytometer, CA). Dead cells were excluded using propidium iodide staining. By utilizing gates for the width and area of forward scattered light, the single cells were selected, followed by gates for the width and area of side scattered light. As soon as the fibroblasts were 80% confluent in the flask, they were collected. We then isolated single living cells following digestion using a fluorescence‐activated cell sorting procedure. One thousand tumor‐associated fibroblasts and 500 tumor organoids were sorted into 48‐well plates of OBM containing 1% Matrigel for co‐culture. Subsequently, the cells were centrifuged at 1000 rpm for 5 min and incubated in a static incubator overnight. A biomatrix was provided for the growth of 3D organoid cells by coating the plastic surface of the wells with Matrigel on the second day. The OEM mice were added when the Matrigel became solid. The diameters of organoids were measured using a scale tool provided by Zen Light Edition Software after 7 days of co‐culturing with cancer‐associated fibroblasts of mouse origin.

### 2.6 Culture in the Trans‐well

Trans‐well cultures involved seeding 1*10^3^ CAF cells over the Trans‐well membrane (1‐μm pore size, 662,610; Greiner Bio‐One) and developing 200 single organoid cells for 14 days in the lowest chamber of 24‐well plates.

### 2.7 Tissue histology, immunohistology, and immunofluorescence

Dissected tissues and organoids were placed in 10% neutral formalin buffer and subsequently embedded in paraffin blocks and serially sectioned with a microtome for subsequent histological analysis. In order to retrieve antigens from paraffin‐embedded tissue sections, pH 6 sodium citrate buffer was used before rehydrating them, to reduce background nonspecific staining, and permeabilise the sample. Using DAKO peroxidase blocker after suppressing endogenous peroxidase, the sections were treated at 4°C overnight with primary anti‐Alpha‐SMA (Abcam, ab124964) and anti‐Epcam (Abcam, Ab71916) antibodies. To assess secondary antibody responses that are not specific, slides were also stained without the presence of primary antibodies. Slides were submerged in 3,3′‐diaminobenzidine (DAB) substrate and allowed to react until the desired color was achieved (30 s to 4 min). Finally, images were then captured using a Zeiss Axioskop microscope after the slides had been counterstained with hematoxylin.

The samples were kept at −80°C. To prepare the tissue for analysis, it was then further dehydrated with 25% sucrose (Sigma‐Aldrich, S0389, overnight at 4°C), then cut into 8‐um sections. A Zeiss LSM510 meta confocal microscope was used to capture the images.

### 2.8 Assay for tumor development using organoids in NSG mice

A tumorigenesis assay was performed in vivo using NSG immunodeficient mice aged 5–6 weeks. The identical mice's two flanks were subcutaneously implanted with 2*10^3^ mouse LGR5^+^ tumor organoid cells, either alone or in combination with 2*10^3^ cancer‐associated fibroblasts. To study whether cancer‐associated fibroblasts promote LGR5^+^ metastasis, we only injected one side of mice with 5*10^3^ isolated LGR5^+^ tumor cells with or without 5*10^3^ cancer‐associated fibroblasts. After 1–2 months, tumors were examined and tumor weight was determined. Mice were kept in a room with a 12‐h light/dark cycle (lights on at 6 a.m.) and free access to food and drink. The Committee of the Tongji University Experimental Animal Center authorized each and every animal experiment.

### 2.9 Statistical analysis

All statistical analyses were performed using Prism software (GraphPad software 8.0). The Mann–Whitney U‐test, which determines whether differences between groups are statistically significant, was used. A *p* value of less than 0.05 was considered significant.

## 3 RESULTS

### 3.1 LGR5 marked tumor cells were located adjacent to cancer‐associated fibroblasts

**FIGURE 1:**
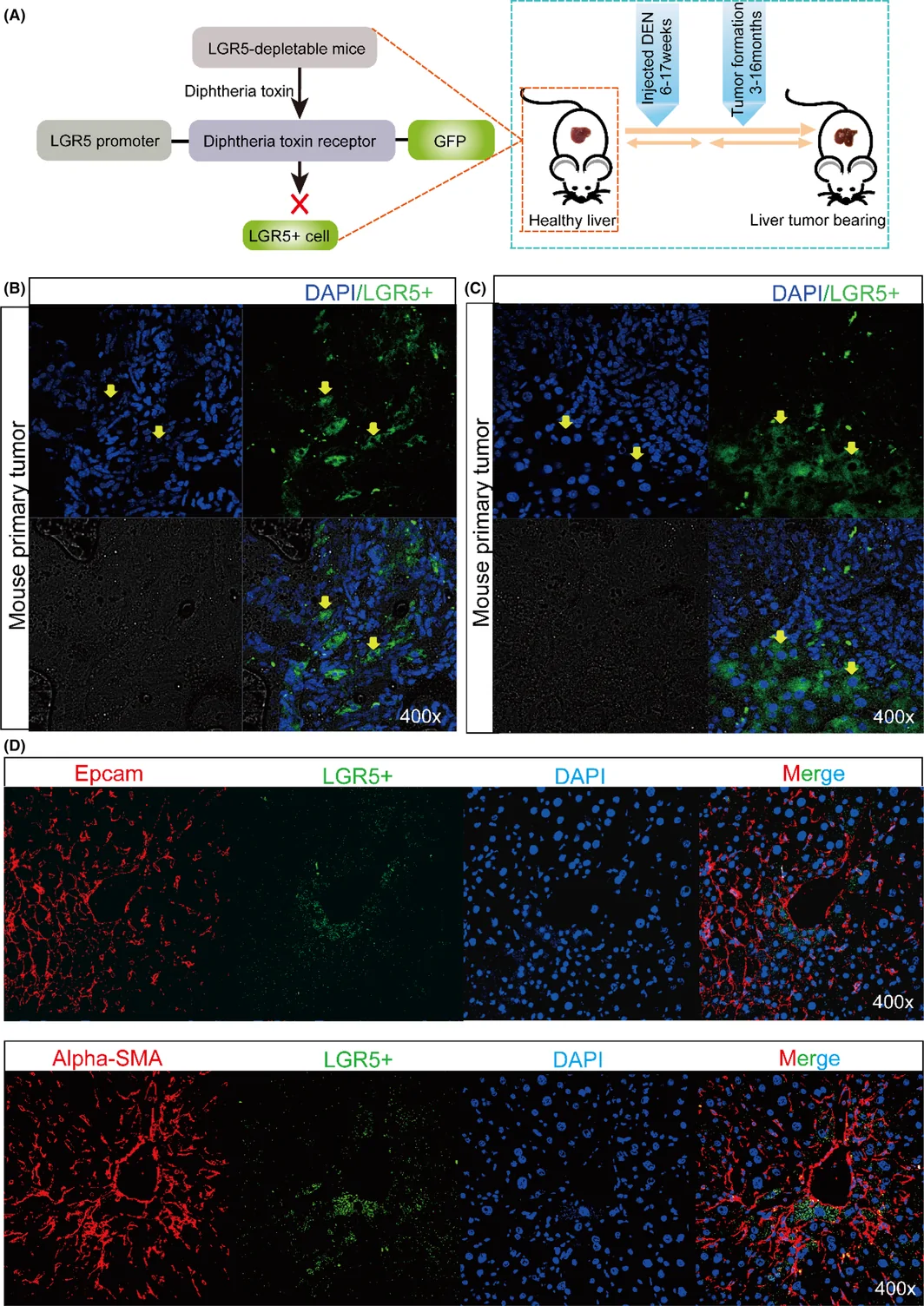
The tumor cells marked by LGR5 were located near the cancer‐associated fibroblasts. (A) Strategy of inducing primary tumor in LGR5–DTR–GFP transgenic mice by diethylnitrosamine (DEN); (B,C) representative images showing LGR5–GFP^+^ cells as present in liver tumors. (Yellow arrow: LGR5–GFP^+^ cell. DAPI: blue), (magnification, 400×); (D) representative images showing LGR5–GFP^+^ cells in primary liver tumors (LGR5‐driven GFP: green, DAPI: blue, Epcam:red, a‐SMA: red, magnification, 400x).

We investigated the detailed relationship between those two cell populations in this manuscript. Thus, LGR5‐GFP‐creERT/Rosa26‐iDTR transgenic mouse model, which allow the direct indication of LGR5 positive expression cells, was initially used to investigate this correlation (Figure 1A). Carcinogen compound diethylnitrosamine (DEN) was administrated in LGR5‐GFP‐creERT/Rosa26‐iDTR transgenic mouse to induce primary murine liver tumor formation. After 6–17 weeks of DEN induction, primary liver tumor formation was observed. Immunofluorescence (IF) staining were further adopted to confirm the tumor formation and evaluate the geographical distribution of LGR5‐GFP^+^ cells in the liver tumors (Figure 1B,C). Very impressively, we found high frequency of CAFs marked by alpha‐smooth muscle actin (α‐SMA) surrounding LGR5 expressing cancer cells in primary liver tumor (Figure 1B–D). In addition, we also further perform the anti‐GFP immunohistochemistry staining, with the intention to visualize the mutual location in a more direct way, we can observe that CAF and LGR5+ cells have a close relationship in position (Figure S1A). In conclusion, we first revealed that the LGR5 marked tumor cells were closely located adjacent to cancer‐associated fibroblasts in DEN‐induced primary murine liver tumor.

### 3.2 Generation and culture of cancer‐associated fibroblasts and tumor organoids from DEN‐induced initial liver tumors in mice

Upon the observation of the close localization of the LGR5 liver tumor cell and CAFs within murine liver cancer, we decided to further establish a three‐dimensional organoid co‐culture system constituting both tumor organoids capable of tracking LGR5 expressing and CAFs from liver tumors, which allow more detailed investigation of their mutual interaction. Thus, we need to separately obtain those two cell populations.

**FIGURE 2:**
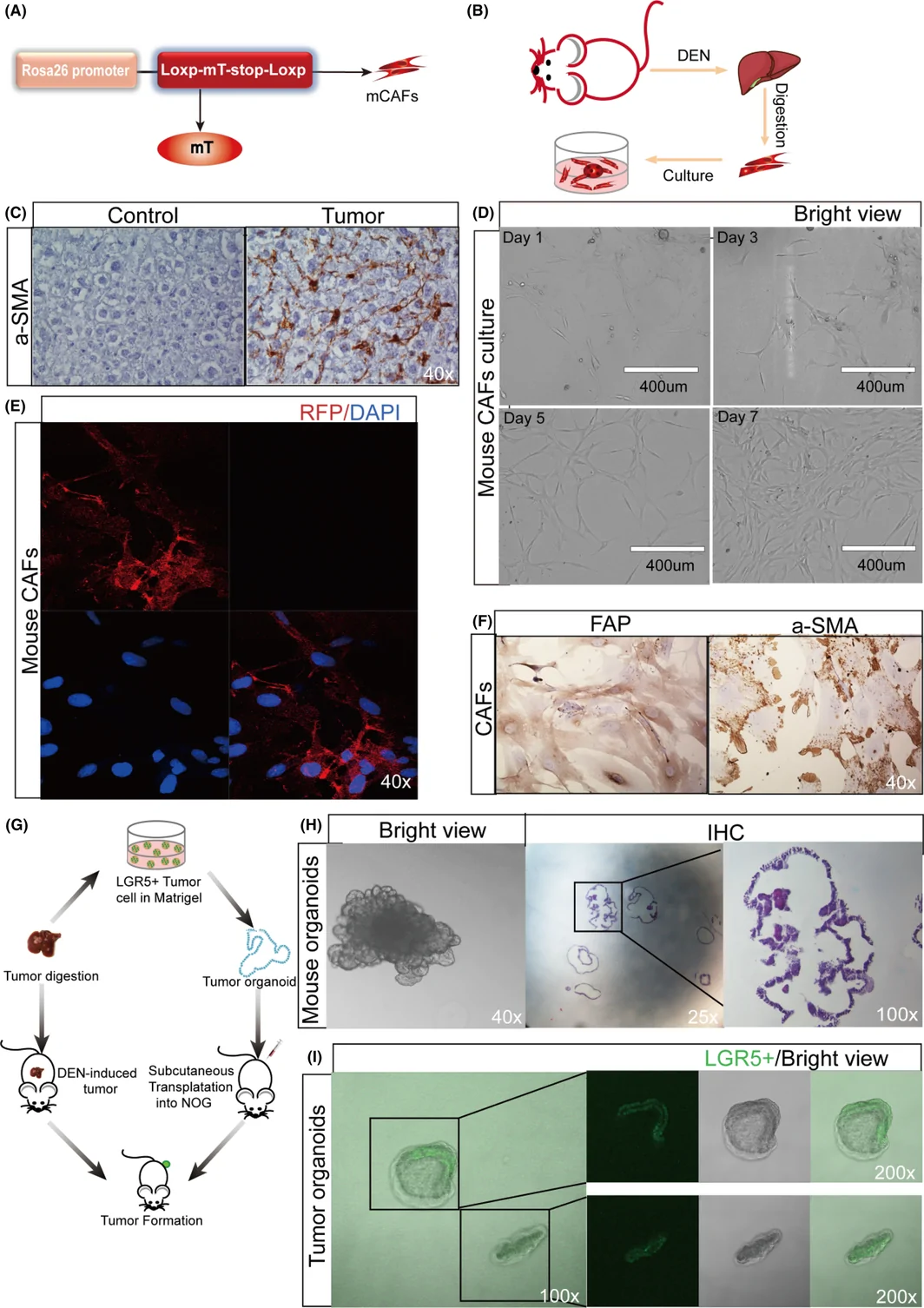
Culture of cancer‐associated fibroblasts and tumor organoids. (A) Construction of Rosa26‐MT transgenic mice expressing red fluorescence; (B) schematic diagram of cancer‐associated fibroblasts culture; (C) representative immunohistochemistry staining of a‐SMA in mouse primary tissue (magnification, 40×); (D) representative image of mouse CAFs cultures from day 0 to day 7 (Scale bar = 50 μm); (E) representative immunofluorescence staining of mouse CAFs (CAF‐driven RFP: red, DAPI: blue, magnification, 40×); (F) representative immunohistochemistry staining of FAP and a‐SMA in mouse CAFs (magnification, 40×); (G) overview of the principle of LGR5‐GFP‐creERT/Rosa26‐iDTR transgenic mice and experimental strategies for organoid establishment; (H) representative images of immunohistochemical staining of liver tumor organoids (Left: magnification, 40×; Middle: magnification, 25×; Inset: magnification, 100×); (I) Representative pictures showing the presence of LGR5‐expressing cells in organoids (magnification, 100×; Inset: magnification, 200×; LGR5‐driven GFP: green. Scale bar = 50 μm).

For the intermediate visualization of CAFs, we isolated this type of cell from Rosa26‐MT‐mice, which all the cells within this mouse model accompanied with expression of red fluorescence (Figure 2A). Carcinogen compound DEN was also administrated in Rosa26‐MT mouse to induce primary liver tumor formation. After 6–17 weeks of DEN induction, primary liver tumor formation was observed (Figure 2B). We observed CAF expressing red fluorescence under a fluorescence microscope (Figure 2E). Immunohistochemistry staining also confirmed cancer‐associated fibroblasts marked by alpha‐SMA (a‐SMA) was highly presented than that of tumor adjacent tissue (Figure 2C). Adopting electron microscope, we observed that CAFs could proliferate during the culture process, showing a long spindle shape, and the spindle shape was further extended after 7 days of culture. (Figure 2D). Immunohistochemical (IH) staining found most cultured CAFs were positive for alpha‐smooth muscle actin (a‐SMA) and fibronectin attachment protein (FAP) (Figure 2F).

For the direct visualization of LGR5 expressing tumor stem cell, murine tumor organoids were cultured from previous DEN‐induced primary liver tumors from LGR5‐GFP‐CreERT/Rosa26‐iDTR transgenic mice (Figure 2G). After 1–4 weeks of culture, murine liver tumor organoids were successfully initiated and can be stably maintained by followed passage (Figure 2H,I, Figure S1B). Additionally, we noticed that the population of LGR5‐positive cells was maintained in the organoids (Figure S1C). Further transplantation of tumor organoids in immunodeficient mice also allowed tumor initiation, which further confirm their malignancy. In sum, both cancer‐associated fibroblasts and tumor organoids which also the direct observation have been successfully generated from the corresponding DEN‐induced mouse primary liver tumors.

### 3.3 Construction a 3D co‐culture model of LGR5 ^+^ expressing tumor cells and cancer‐associated fibroblasts in direct contact and Trans‐well system

For establishment of co‐culture model, we also further investigated whether the organoid culture medium also suitable for the culture of CAFs. Four different media, including organoid initiate medium, organoid basic medium, organoid reduced medium (which removed B27 and N‐acetylcysteine due to their impact on proliferation of CAFs), and RPMI 1640 buffer, were separately adopted to culture CAFs (Table S1). And their cell viability was monitored. We found that the proliferation of CAFs cultured in organoid initiate medium and organoid reduced medium was significantly higher than that in the other two medium (Figure S2A); thus, initiate medium and reduced medium were adopted in the following coculture system.

**FIGURE 3:**
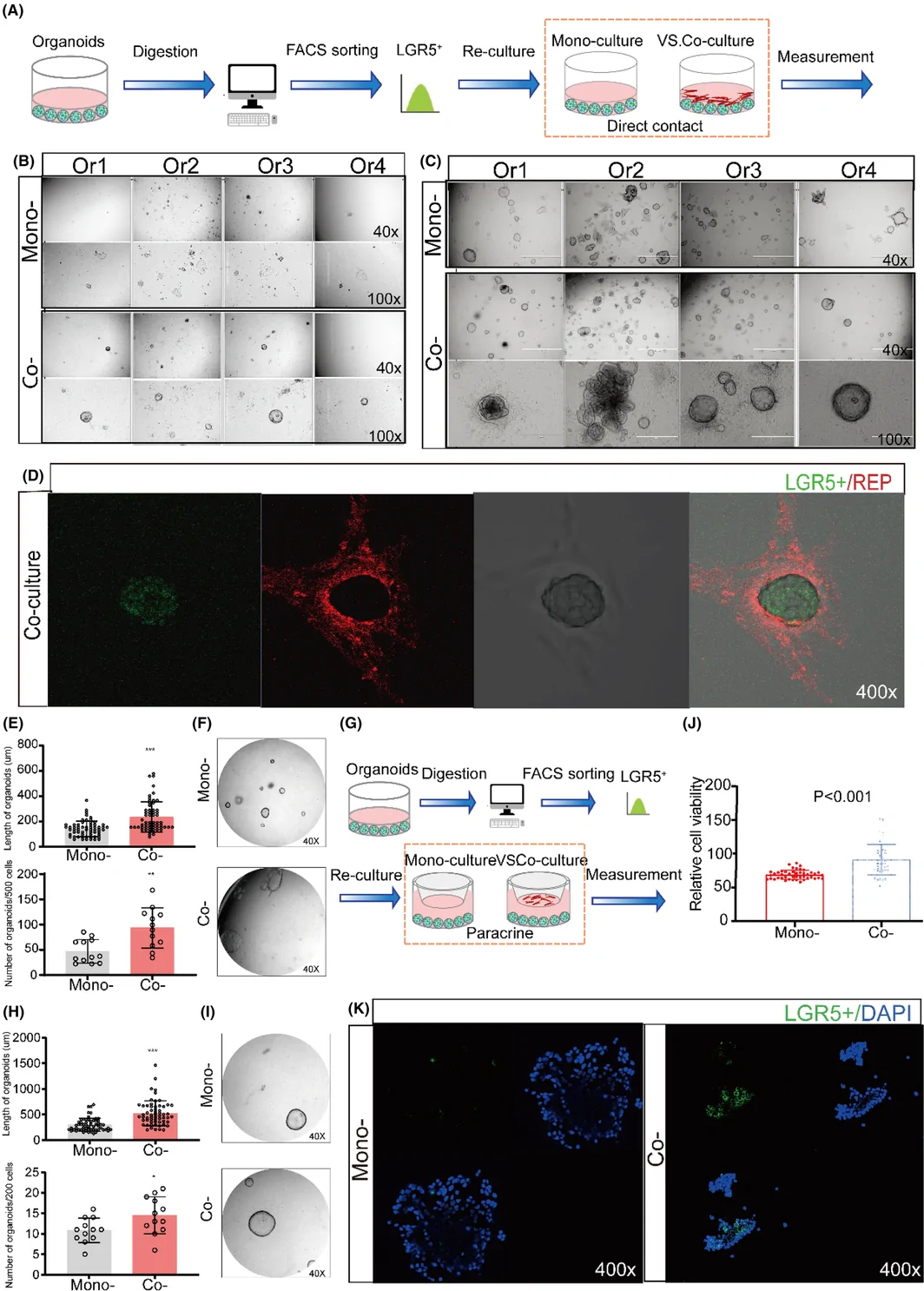
Establishment of organoid and CAF co‐culture models of mouse. (A) LGR5^+^ mono‐culture and LGR5^+^+cancer‐associated fibroblasts direct contact co‐culture experimental strategies; (B) LGR5+ mono‐ and LGR5^+^+CAFs co‐culture on day 1 (*n* = 4); (C) LGR5 + mono‐ and LGR5^+^+cancer‐associated fibroblasts co‐culture on day 5 (*n* = 4); (D) representative confocal image of mouse organoids and CAF co‐culture model (CAF‐driven RFP: red, LGR5‐driven GFP: green, magnification, 400×); (E) diameters and number of tumor organoids cultured with or without CAFs (*n* = 4 experimental settings with three biological replicates for each. Five organoids for each well were randomly measured, Mann–Whitney U‐test, *p < 0.05, **p < 0.01, ***p < 0.001); (F) representative images of mono‐, co‐cultured organoids; (G) experimental strategies of LGR5^+^ mono‐culture and LGR5^+^+CAFs paracrine co‐culture; (H) diameters and number of organoids in trans‐well platform with or without CAFs (*n* = 4 experimental settings with 3 biological replicates for each. Five organoids for each well were randomly measured, Mann–Whitney U‐test, *p < 0.05, **p < 0.01, ***p < 0.001); (I) Representative images of mono‐, co‐cultured organoids in a trans‐well system. (J) Growth of mouse organoids determined by Alamar Blue Assay *p < 0.05, **p < 0.01, ***p < 0.001); (K) Representative images of LGR5 express of LGR5^+^ mono‐culture and LGR5^+^+CAFs paracrine co‐culture (LGR5‐driven GFP: green, DAPI: blue, magnification 400×).

Subsequently, the 3D co‐culture organoid system was followed to construct with the intention to investigate the interaction between CAFs and LGR5^+^ expressing tumor cells. First, tumor organoids isolated from LGR5‐GFP‐creERT/Rosa26‐iDTR transgenic mice were digested into single cells and LGR5‐expressing tumor cells were sorted out by flow cytometry based on GFP expression. And then, those cells were culture on the top of a layer of matrigel first and followed by seeding of red florescent expressing CAFs (Figure 3A–C). We observed that LGR5‐expressing tumor cells will grow into organoid which surrounded closely by CAFs, within the direct cell–cell contact co‐culture model. We confirmed that CAFs firmly encircled LGR5^+^ tumor cells using immunofluorescence staining. (Figure 3D). The size and number of organoids formed by in vitro cell–cell contact co‐culture were significantly increased as compared with LGR5^+^ cells cultured alone (Figure 3B,E,F).

In addition, transwell culture was adopted to investigate the indirect interaction. On top of the Transwell membrane, CAF cells were seeded. In the lower compartment, single LGR5‐expressing tumor cells are growing for 10 days (Figure 3G). The similar results of the size and number of organoids formed were significantly increased as compared with LGR5^+^ cells cultured alone within the transwell system of paracrine signaling between LGR5^+^ tumor cells and cancer‐associated fibroblasts (Figure 3H,I). At the same time, LGR5^+^ cells or CAFs with LGR5^+^ cells incubated medium collected from co‐cultures was used to analyze the metabolic activity of the cells. Alamar Blue assay confirmed the relative cell viability of co‐cultures was notably higher than that of LGR5^+^ cells single cultures (Figure 3J). Notably, co‐culture promoted cell proliferation and LGR5 expression (Figure 3K). Based on these results, we concluded that CAFs could accelerate the organoid initiation and growth of LGR5^+^ single cells both in direct contact and Trans‐well co‐culture systems.

### 3.4 Cancer‐associated fibroblasts can promote the growth of tumor and the expression of LGR5 in mice

**FIGURE 4:**
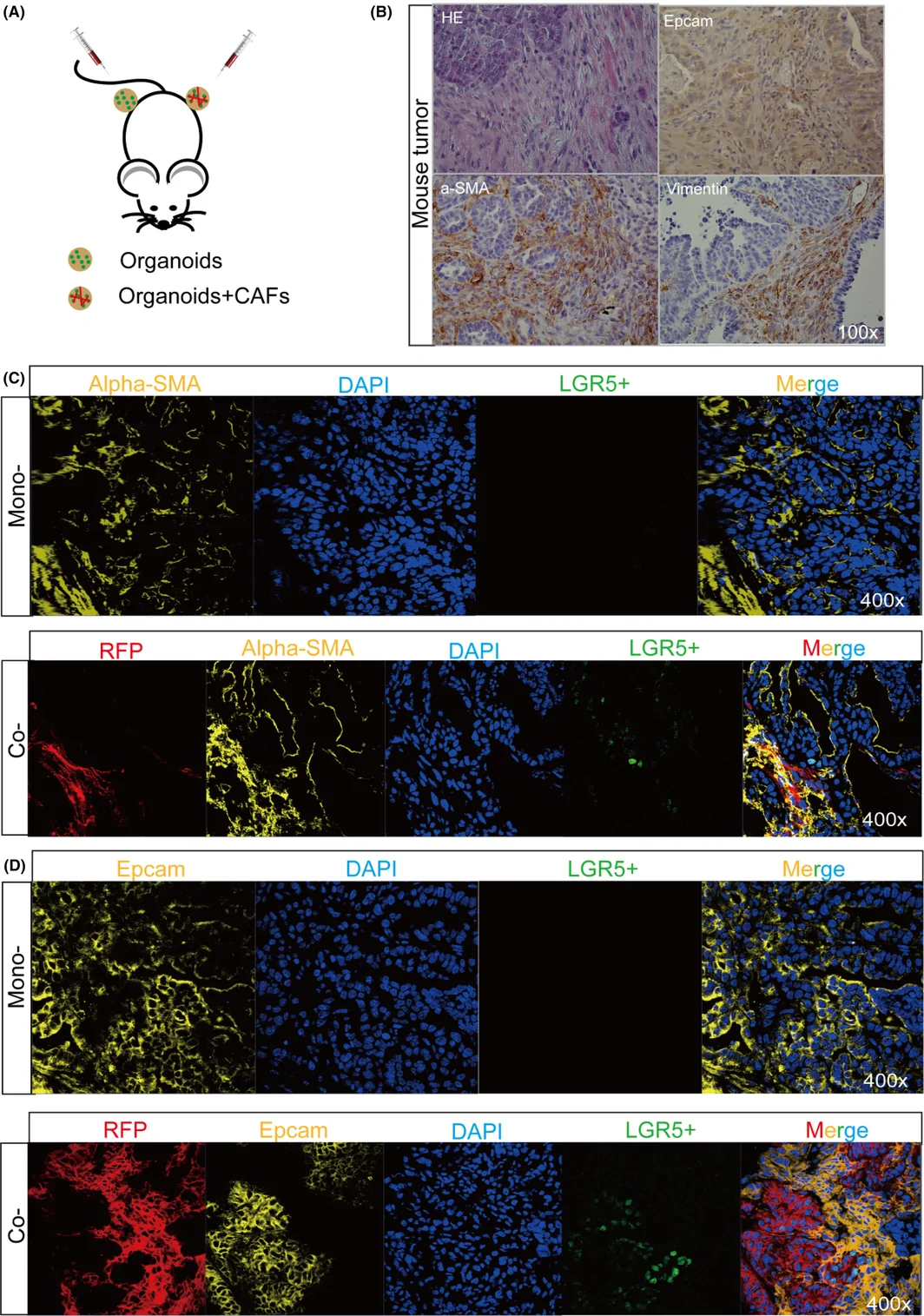
Cancer‐associated fibroblasts and organoids subcutaneously engraft promoted tumor growth and LGR5 expression in mice. (A) 2.5*10^5^ mouse liver tumor organoid cells with or without 2.5*10^5^ mouse CAFs were subcutaneously engrafted into immunodeficient mice; (B) the representative immunohistochemistry staining of H&E, Epcam, a‐SMA, and Vimentin for tumors from co‐transplantation (magnification, 100×); (C,D) representative confocal images of LGR5 expression in tumors from mono‐ or co‐engraftment (LGR5‐driven GFP: green, DAPI: blue, CAF‐driven RFP: red, Epcam: red, a‐SMA: red, magnification 400x).

Previous results have proved that CAFs can promote the growth of LGR5‐expressing cell to initiated tumor organoids and maintain their structure in an in vitro 3D culture system. We further investigated the mutual interaction of LGR5‐expressing tumor cell and CAF in vivo within immunodeficient mouse model. We subcutaneously implanted organoids alone and the mixture of tumor organoid with CAFs cells in the 1:1 ratio separately on the different location of the same immunodeficient mice. (Figure 4A). Tumors were successfully initiated after 7 days. Following tumor collection, immunohistochemistry was used to establish the presence of CAFs in the tumor tissues of co‐transplanted animals (Figure 4B, Figure S1D). After analysis, the tumor volume of mice in organoid +CAFs co‐implantation group was significantly larger than mono‐engraftment group (Figure S1E). We also found that co‐engraftment of liver tumor organoids with CAFs resulted in dramatic higher number of LGR5^+^ cells in the formed tumors when compared with engrafting tumor organoids alone (Figure 4C,D). This is consistent with the precious results of in vitro co‐culture. Hence, the existence of CAFs could promote the growth of tumors and enhance the expression number of LGR5 expressing cells in xenograft murine tumor.

### 3.5 Co‐culture of cancer‐associated fibroblasts derived from co‐engraftment tumors with LGR5+ cells further enhanced organoid growth

**FIGURE 5:**
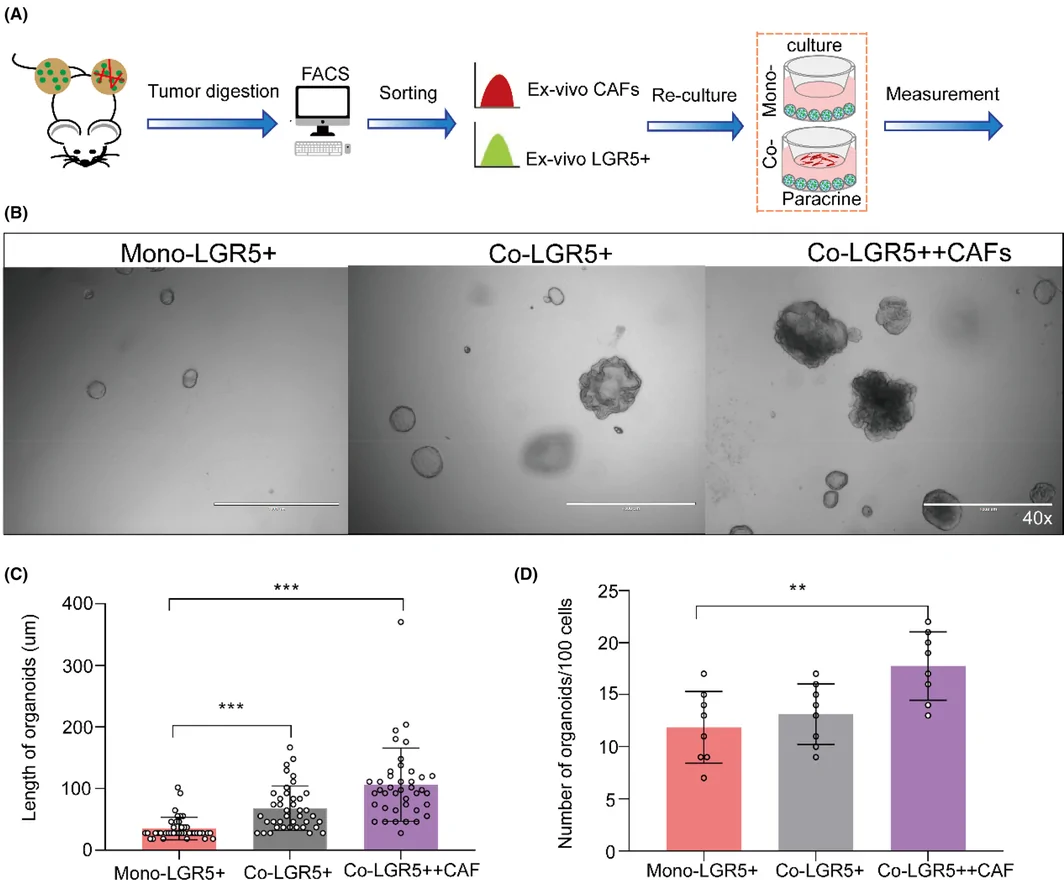
Co‐culture of cancer‐associated fibroblasts derived from co‐engraftment tumors with LGR5^+^ cells further enhanced organoid growth. (A) An outline of the experimental strategy used to perform ex vivo mono‐ and co‐culture; (B) representative picture of ex vivo cultured LGR5^+^ cells with or without CAFs from mono‐ or co‐engraftment tumors (magnification 40×); (C) diameters of ex vivo cultured LGR5^+^ cells with or without CAFs (*n* = 4 experimental settings with 2 biological replicates for each. Five organoids for each well were randomly measured, *p < 0.05, **p < 0.01, ***p < 0.001); (D) Number of ex vivo cultured LGR5^+^ cells with or without CAFs (*n* = 4 experimental settings with 2 biological replicates for each, *p < 0.05, **p < 0.01, ***p < 0.001).

In our previous study, we demonstrated that CAFs could boost the growth of tumor organoids and enhance the percentage of LGR5 expressing cells both in vitro co‐culture system and in vivo mice model. Thus, we further explored the influence of CAFs which directly isolated from tumors to LGR5‐expressing tumor organoid. CAFs traced by red fluorescent protein (RFP) were isolated from co‐engraft tumors and GFP‐expressing LGR5‐expressing tumor cells were separated from both mono‐ and co‐engraft tumors (Figure 5A). Ex vivo culture of isolated LGR5^+^ cells from tumors of co‐engrafted mice formed significantly larger size of organoids than isolated mono‐engrafted (68.1 ± 36.1 vs. 35.2 ± 18.3 μm, *n* = 8, each well was measured with five organoids at random, *p* < 0.001), although there is no statistically significant difference in the number of formed organoids (Figure 5B–D). Those two types of cells were further culture both in direct and indirect method to investigate the mutual influence. We found that co‐culture of LGR5^+^ cells with ex vivo obtained remnant CAFs from tumors of co‐engrafted mice also can further promote the size and number of formed organoids (Figure 5B–D). Therefore, we concluded that co‐culture of CAFs with LGR5^+^ cells isolated from co‐engraft tumors could promote organoid growth.

### 3.6 Cancer‐associated fibroblasts nurture LGR5‐marked liver tumor‐initiating cells and promoted their metastasis

**FIGURE 6:**
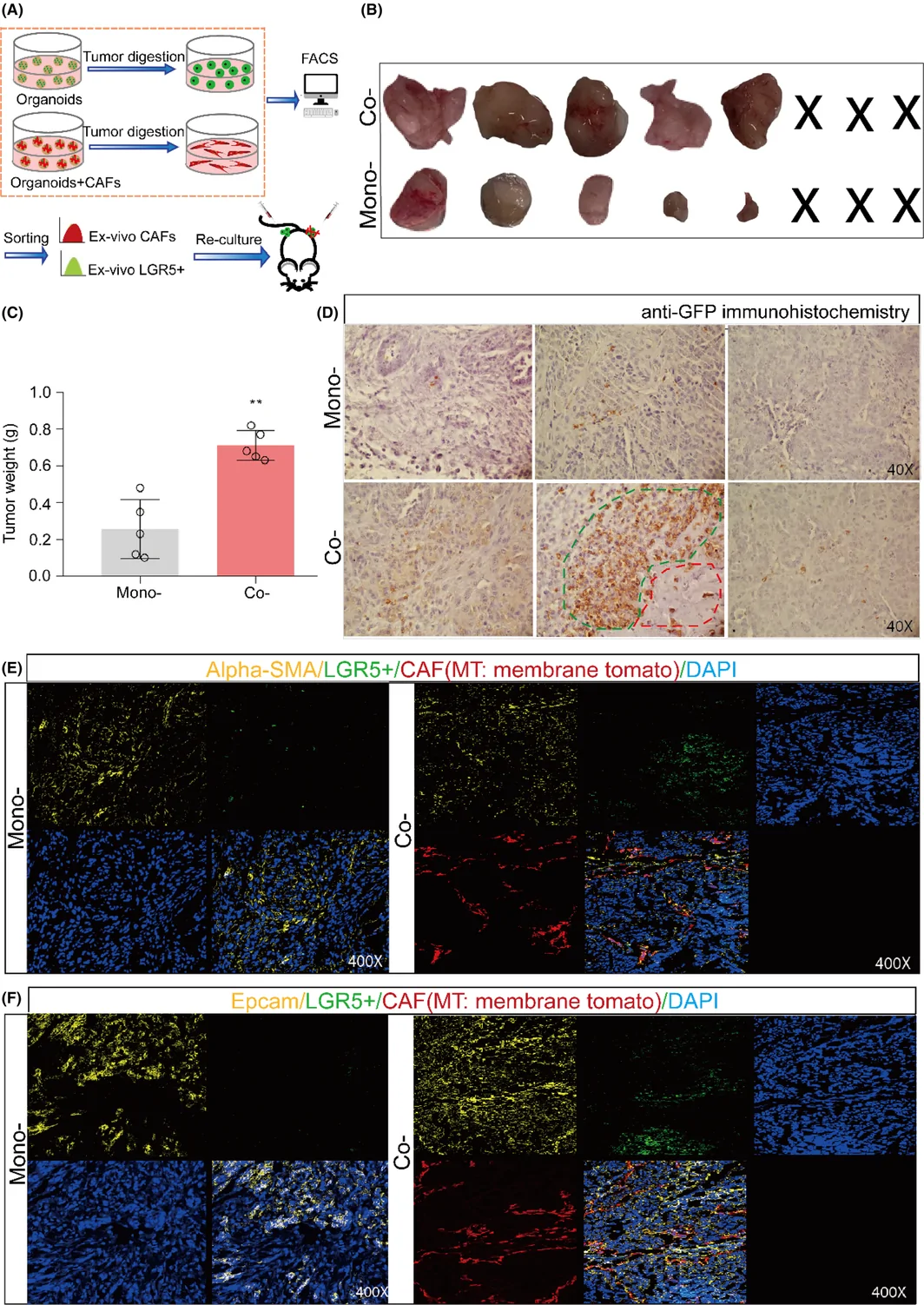
CAFs promoted the growth and metastasis of LGR5‐marked liver tumors. (A) 2*10^3^ isolated LGR5^+^ tumor cells with or without 2*10^3^ CAFs were engrafted into immunodeficient mice; (B) pictures showed the tumors from mono‐ and co‐engraftment; (C) the weight of tumors from mono‐ or co‐engraftment (*n* = 5, Mann–Whitney U‐test, *p < 0.05, **p < 0.01, ***p < 0.001); (D) the representative immunohistochemistry staining of anti‐GFP for tumors from mono‐ (LGR5^+^ cells) or co‐engraftment (LGR5^+^ cells with CAFs) (*n* = 3, LGR5‐driven GFP: green, CAF: red, magnification 40×); (E,F) representative confocal image of LGR5 expression for tumors from LGR5^+^ cells mono‐ or LGR5^+^ cells with CAFs co‐engraftment (LGR5‐driven GFP: green, DAPI: blue, CAF‐driven RFP: red, Epcam: red, a‐SMA: red, magnification 400x).

Consistently, we further to investigate the influence of CAFs to LGR5‐expressing cell regarding the tumor initiation and growth. Single LGR5‐expressing cells were sorted out and further mixed with CAFs, followed by subcutaneously co‐engraftment into immunodeficient mice (Figure 6A).

After tumor initiation, we found that the supplement of CAFs with LGR5^+^ tumor cells formed significantly larger tumors compared with engrafting LGR5^+^ cells alone (0.6 ± 0.2 vs. 0.3 ± 0.2 g, *n* = 5, *p* < 0.05, Figure 6B,C). Immunohistochemical staining of anti‐GFP indicated an obvious high LGR5 expression in co‐engraftment (*n* = 3) (Figure 6D). To be noted, co‐engraftment maintained a high‐expressing LGR5^+^ population that are close to remnant CAFs (Figure 6E,F).

Meanwhile, we found that three out of eight mice died likely attributed to massive colon, spleen or liver metastasis. Inversely, the corresponding transplanted tumors formed on the two sides of original engraftment still remain small size surprisingly (Figure S1F). Interestingly, these metastatic tumors harbor cells highly expressing LGR5 (Figure S1G), indicating the transplanted cell‐related metastasis. In conclusion, we suggest that co‐implantation of LGR5^+^ tumor cells with cancer‐associated fibroblasts could increase tumor size and promote further metastasis.

### 3.7 Depletion of the LGR5 lineage by DT inhibited murine organoid and tumor growth and metastasis

**FIGURE 7:**
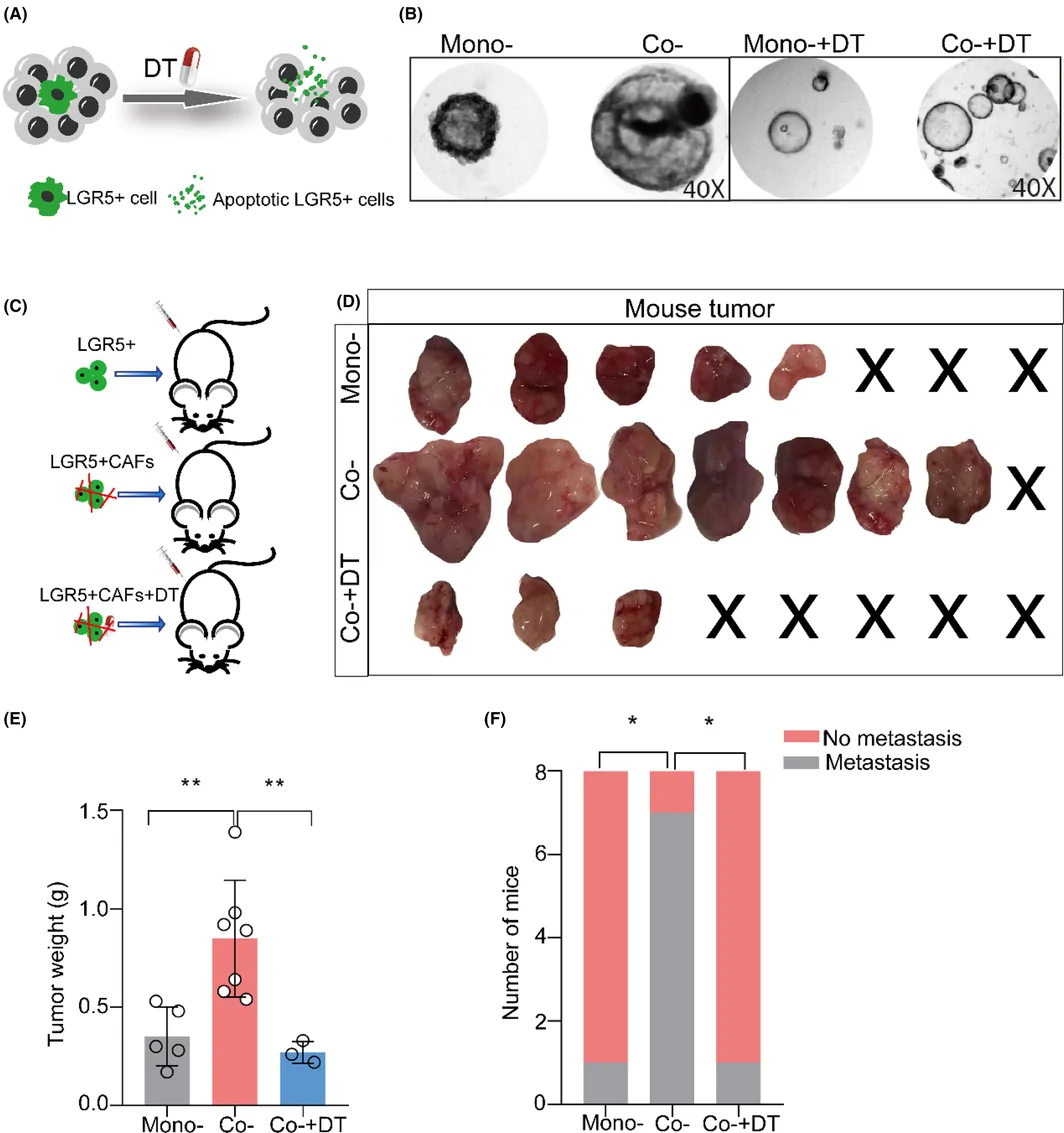
LGR5 depletion inhibits tumor growth and metastasis. (A) Outlines of the experimental strategy used to assess the efficacy of DT administration on allograft tumors in mice; (B) representative picture of LGR5^+^ cells mono‐culture, co‐culture with CAFs, mono‐culture plus DT and co‐culture plus DT treatment (magnification 40×); (C) subcutaneous engraftment of 5*10^3^ LGR5^+^ cells with or without 5*10^3^ CAFs, and DT treatment in the co‐engrafted mice; (D) tumor pictures showed the tumors from mono‐, co‐engraftment with or without DT treatment; (E) the weight of tumors from mono‐, co‐engraftment with or without DT treatment (*n* = 5 for mono‐engraftment, *n* = 7 for co‐engraftment, *n* = 3 for co‐engraftment with DT treatment, Mann–Whitney U‐test, *p < 0.05, **p < 0.01, ***p < 0.001); (F) number of abdominal metastasis for the three groups (*n* = 8, Chi‐square test, *p < 0.05, **p < 0.01, ***p < 0.001).

Based on the aforementioned findings, we deduced that the liver cancer progression will be impacted upon the ablation of LGR5^+^ cells even with the presence of CAFs. For the purpose of testing the hypothesis, we utilized the co‐expression of the diphtheria toxin receptor (DTR) in LGR5‐GFP‐CreERT/Rosa26‐iDTR mice. Due to the DT‐DTR‐specific depletion system, we would be able to specifically deplete the LGR5‐expressing cells by administration of diphtheria toxin (DT) (Figures 1A and 7A). Thus, we cultured LGR5^+^ cells alone or co‐cultured with cancer‐associated fibroblasts, under the situation with/without DT supplement to ablate LGR5‐expressing cells. Then, we found this tumor growth promoting effect was dramatically eliminated by DT treatment upon specific depletion of LGR5‐expressing cells (Figure 7B).

To clarify whether the previous observed metastasis was promoted by CAFs, we established three individual groups in immunodeficient mice, subcutaneously engrafting isolated LGR5^+^ tumor cells, LGR5^+^ cells with CAFs and LGR5^+^ cells with CAFs with DT administration (Figure 7C). Consistently, co‐engraftment facilitated the formation and growth of tumors at the original location, but this promoting effect was eliminated by DT treatment which specifically depletes LGR5^+^ cells (Figure 7D,E). Most importantly, co‐engraftment dramatically increased the probability of abdominal metastasis (7/8) compared with mono‐engraftment of LGR5^+^ tumor cells (1/8). However, DT treatment dramatically reduced the probability of metastasis in this xenograft model (Figure 7F). In sum, we concluded that specifically depletion of the LGR5 lineage by DT administration inhibited murine organoid and tumor growth and metastasis, which promoted by mutual influence of CAFs and LGR5‐expressing tumor stem cells.

## 4 DISCUSSION

Liver cancer, in particular hepatocellular carcinoma, commonly metastasizes to lung, lymph node, and bone with poor prognosis. However, the exact tumor cell populations that are responsible for metastasis remain largely unknown. Previous finding that defined liver LGR5^+^ tumor cells as tumor initiating cell population.^8^ We extend previous studies and further demonstrate that these cells are capable of metastasis in mouse model. This, however, heavily relies on the nurturing effects of CAFs. In recent years, the research on targeted stem cell therapy has received extensive attention. Junttila and colleagues report an interesting approach to the use of antibody‐drug conjugates (ADCs) targeting LGR5^+^ cells to treat colon cancer.^21^ It has also been reported that two antigen‐drug conjugates (ADCs) targeting LGR5 have been prepared by linking the cytotoxic drug mono‐methyl auristatin E, which inhibits tubulin, to a highly specific monoclonal antibody against LGR5 through a proteolytic cleavable or non‐cleavable chemical linker. Anti‐LGR5 ADC has obvious killing effect on gastrointestinal cancer cells with high expression of LGR5, but has no obvious killing effect on gastrointestinal cancer cells with negative or knockdown of LGR5.^22^

However, current published research showed very limited consideration related to the influence of tumor microenvironment to LGR5 tumor stem cell related tumor treatment. Researcher already revealed healthy LGR5 stem cell has been revealed that nourished by fibroblast. It has been shown that LGR5^+^ cells in the basal and lower isthmus of the gastric gland and adjacent highly proliferative LGR5^−^ cells regulate the regeneration of the gastric epithelium by expressing Axin2.^23^ In addition, expression of Axin2 and LGR5 requires matrix‐derived R‐spine 3 produced by gastric myofibroblasts proximal to the stem cell compartment.^24^ Meanwhile, the interaction between tumor LGR5 stem cells and tumor fibroblasts is also under investigation, but the mechanism of action is still unclear and needs further investigation.

In this study, we pioneeringly investigated the mutual relationship between tumor fibroblast cell and LGR5‐expressing tumor stem cell, by constructing an innovated co‐culture model constituting of CAF and LGR5^+^ cells, which allow the culture of both cell types. We found that co‐implantation of LGR5^+^ tumor cells and CAFs increased tumor size. The corresponding mechanism needs to be further investigated in the following research. According to studies that have been published, cancer‐associated fibroblasts are important elements of the tumor microenvironment that can communicate with tumor cells directly and secrete a complex proteome that includes chemokines, extracellular matrix proteins, matrix metalloproteinases, and cytokines inhibit immune cell function and promote tumor development.^27^ CAFs play a prominent role in shaping the stem cell niche to nurture TICs.^28^ According to published studies, the specific cytokines secreted by CAFs include TGF‐β, platelet‐derived growth factor, fibroblast growth factor, hepatocyte growth factor, vascular endothelial growth factor, TNF‐α, INF‐α, CXCL12, IL‐6, IL‐33, etc. Among them, IL‐6 and IL‐33 regulate MDSCs (myeloid‐derived suppressor cells) to inhibit cytotoxic T cells to achieve an optimal microenvironment for enhancing tumor stemness. Vascular endothelial growth factor (VEGF) can regulate tumor vascular network. TGF‐β inhibited DC maturation and promoted Treg differentiation. CXCL12 up‐regulate the anti‐apoptotic proteins Bcl‐2 and Survivin in tumor cells, resulting in drug resistance.^29^, ^30^, ^31^, ^32^, ^33^ The ability of CAFs to keep crosstalk between several signaling pathways active by secreting necessary components is credited with explaining their multifunctional role.

This study found that CAFs nurture LGR5‐marked liver TICs and promoted their metastasis, but the origin of CAFs is not clear, so their properties are not fully characterized. They might originate from epithelia, mesenchymal stem cells, adipocytes, resident fibroblasts, and bone marrow stem cells.^34^ However, current studies have shown that the effect of cancer‐associated fibroblasts on LGR5^+^ stem cells varies in different studies. For instance, αSMA^+^ cancer‐associated fibroblasts in colorectal cancer (CRC) have been shown to exert tumor‐restraining functions by suppressing LGR5^+^ TICs and promoting anti‐tumor immunity.^35^ Another study in CRC reported that LGR5^+^ and LGR5^−^ cancer cells can give rise to liver metastasis. However, LGR5+ cells can lose stemness once outside the stem cell niche and thereby convert to a LGR5‐state.^36^ Thus, the mechanism by which CAFs cultivate LGR5‐labeled liver TICs and promote their metastasis needs to be further studied.

Most importantly, we discovered that LGR5+ cells were specifically depleted in experimental liver cancer models, inhibiting CAF‐mediated tumor formation, growth, and metastasis. Therefore, the interaction between CAFs and LGR5^+^ cells maybe closely related to the LGR5 tumor stem cell‐related liver tumor initiation or development liver cancer. Further validation is required in clinical setting, understanding the intimate interactions of TICs with CAFs shall help to decipher the mechanistic insight of liver cancer progression and develop effective treatment for reducing tumor burden and preventing metastasis.

Recent studies have found that targeting CAF‐mediated immunosuppression may sensitizing tumors to immunotherapy, which provide a potential therapeutic approach for clinical melanoma treatment.^37^, ^38^ Previously, we mentioned that targeting LGR5 can treat gastrointestinal tumors. Therefore, we believe that targeting LGR5 and CAF is a new therapeutic idea. In addition, in the following research, we goanna further investigate the possible combination treatment between the microenvironment adjustment‐related therapy/cancer fibroblast cell targeted therapy with the stem cell‐related therapeutic treatment more comprehensively, which will definitely help to give more viable suggestion for the further clinical usage.

From the aforementioned information, we were able to successfully create a 3D co‐culture organoid model made up of CAFs and LGR5+ tumor‐initiating cells from mouse liver tumors. Our results indicated significantly larger size and number of formed organoids, when compared with culturing LGR5^+^ cells alone in both cell–cell contact and paracrine signaling in vitro. Furthermore, we found that specific knockout of LGR5‐expressing cells suppressed CAF‐mediated promotion of oncogenesis, growth, and metastasis in the experimental mouse liver model. There were some limitations in the present study. First, further research is required to determine the mechanism by which CAFs grow LGR5‐labeled liver TICs and encourage their spread. Second, the origin of CAFs is not well understood, so their properties are not fully defined. Additionally, the close interactions between TICs and CAFs will assist to unravel the mechanistic insight into the genesis of liver cancer and create effective treatments for lowering tumor burden and avoiding metastasis. Further validation in the clinical context is necessary.

## AUTHOR CONTRIBUTIONS

**Mingna Zhang:** Conceptualization (lead); data curation (lead); formal analysis (equal); methodology (lead); software (lead); validation (equal); visualization (equal); writing – original draft (lead). **Yiqiao Fang:** Conceptualization (lead); data curation (lead); formal analysis (equal); methodology (lead); software (equal); validation (equal); visualization (equal); writing – original draft (equal). **Xia Fu:** Conceptualization (lead); data curation (lead); formal analysis (equal); methodology (lead); software (lead); validation (equal); visualization (equal); writing – original draft (equal). **Jiaye Liu:** Writing – review and editing (equal). **Yang Liu:** Investigation (lead); methodology (lead). **Zhounan Zhu:** Investigation (lead); methodology (lead). **Yinyun Ni:** Investigation (lead); methodology (lead). **Menglin Yao:** Investigation (lead); methodology (lead). **Qiuwei Pan:** Writing – review and editing (equal). **Wanlu Cao:** Writing – review and editing (equal). **Zhihui Li:** Conceptualization (lead); data curation (lead); funding acquisition (lead); writing – original draft (lead); writing – review and editing (lead). **chunyan Dong:** Conceptualization (lead); data curation (lead); funding acquisition (lead); investigation (lead); methodology (lead); project administration (lead); writing – original draft (lead); writing – review and editing (lead).

## FUNDING INFORMATION

This work was supported by the National Natural Science Foundation of China (82203861,82073387, 82003283,21671150), the Science and Technology Commission of Shanghai Municipality (14DZ2261100, 15DZ1940106), the fellowship of China Postdoctoral Science Foundation (2021 M702340), Sichuan Science and Technology Program (2021ZYCD016, 2020YFS0573, 2022NSFSC1441), Key Research and Development Program of Science and Technology Department of Sichuan Province 2019YFS0360. Q.P. was supported by the Dutch Cancer Society with a KWF Young Investigator Grant (10140).

## CONFLICT OF INTEREST STATEMENT

The authors declare that the research was conducted in the absence of any commercial or financial relationships that could be construed as a potential conflict of interest.

## ETHICAL APPROVAL STATEMENT

All of the animal experiments in this work have received animal ethical permission (Ethics No. TJBB00722111) from the Tongji University Experimental Animal Center, Shanghai, China.

## Supporting information


Data S1.

## Data Availability

The source data for this study are available from the corresponding author upon reasonable request.
