# Supplementary material for: Cancer‐associated fibroblasts nurture LGR5 marked liver tumor‐initiating cells and promote their tumor formation, growth, and metastasis
Source: Cancer Med. 2023 Aug 14;12(17):18032–49. doi: 10.1002/cam4.6408 (PMC10524013; doi:10.1002/cam4.6408)
Supplement: Supplementary file 1 — Data S1. [file CAM4-12-18032-s001.docx]

# Supplementary Figures and Tables

# Supplementary Figure 1


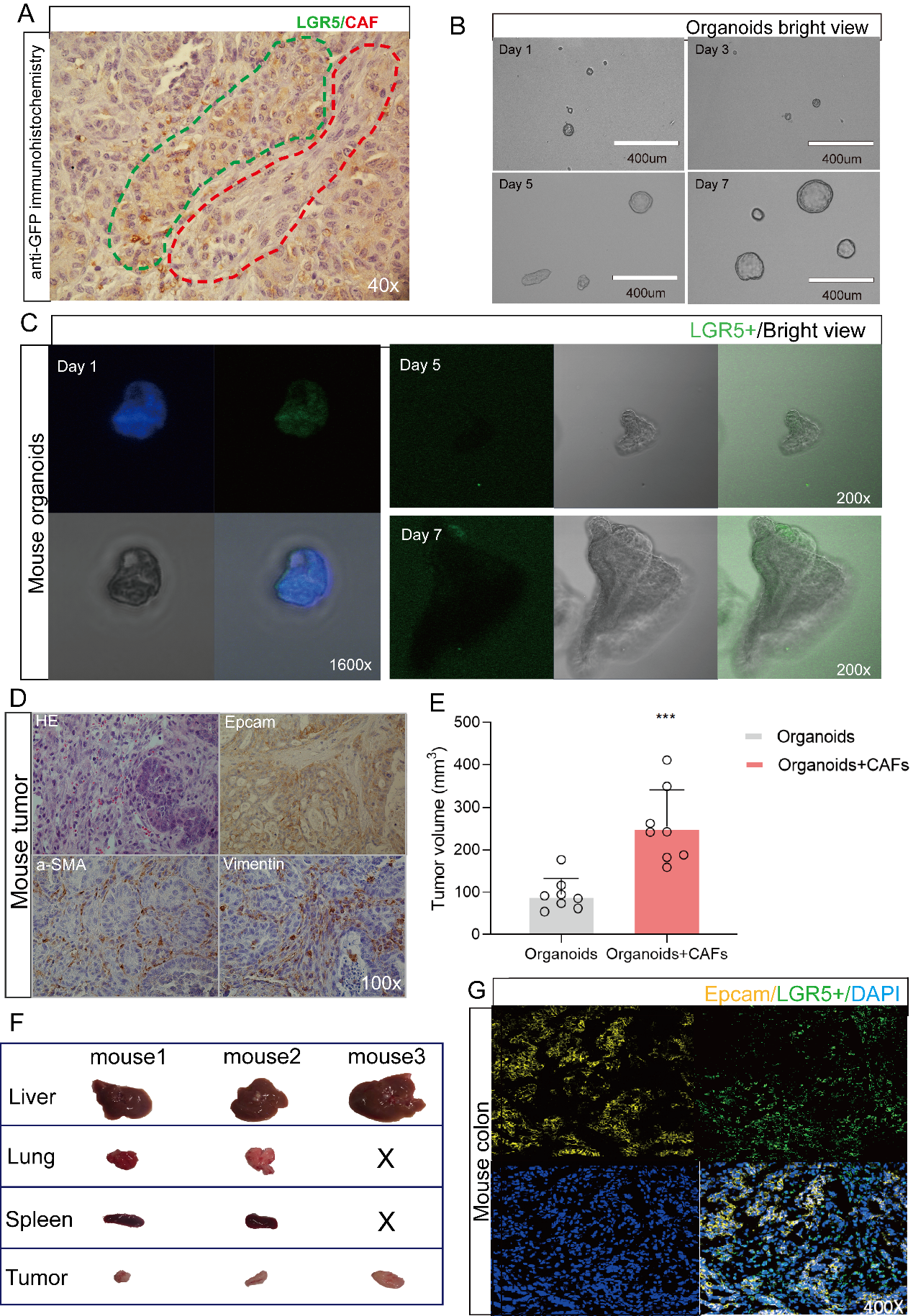


SFigure 1: Cancer-associated fibroblasts nurture LGR5 marked liver tumor-initiating cells and promote their metastasis. （A） The representative immunohistochemistry staining of anti-GFP in primary liver tumors (n = 3, LGR5-driven GFP: green, CAF: red, magnification 40x); (B) Representative image of murine organoids cultures from day 0 to day 7 under ordinary microscope (Scale bar = 50 µm); (C) Representative images of murine organoids from day 0 to day 7 under fluorescence microscopy (LGR5-driven GFP: green, magnification, 200x); (D) The representative immunohistochemistry staining of H&E, Epcam, a-SMA, and Vimentin for tumors from co-transplantation (magnification, 100x); (E) Organoids mono-engraftment group and organoids +CAFs co-engraftment group mice statistical plots of tumor volumes (n = 8, Mann Whitney U test, *P < 0.05, **P < 0.01, ***P < 0.001); (F) Representative images of organs, tumor metastases and corresponding tumor; (G) Representative confocal image of LGR5 expression from colon metastasis of the co-engraftment mice (Epcam: yellow, LGR5-driven GFP: green, DAPI: blue, magnification, 400x).

**Supplementary Figure 2**

**
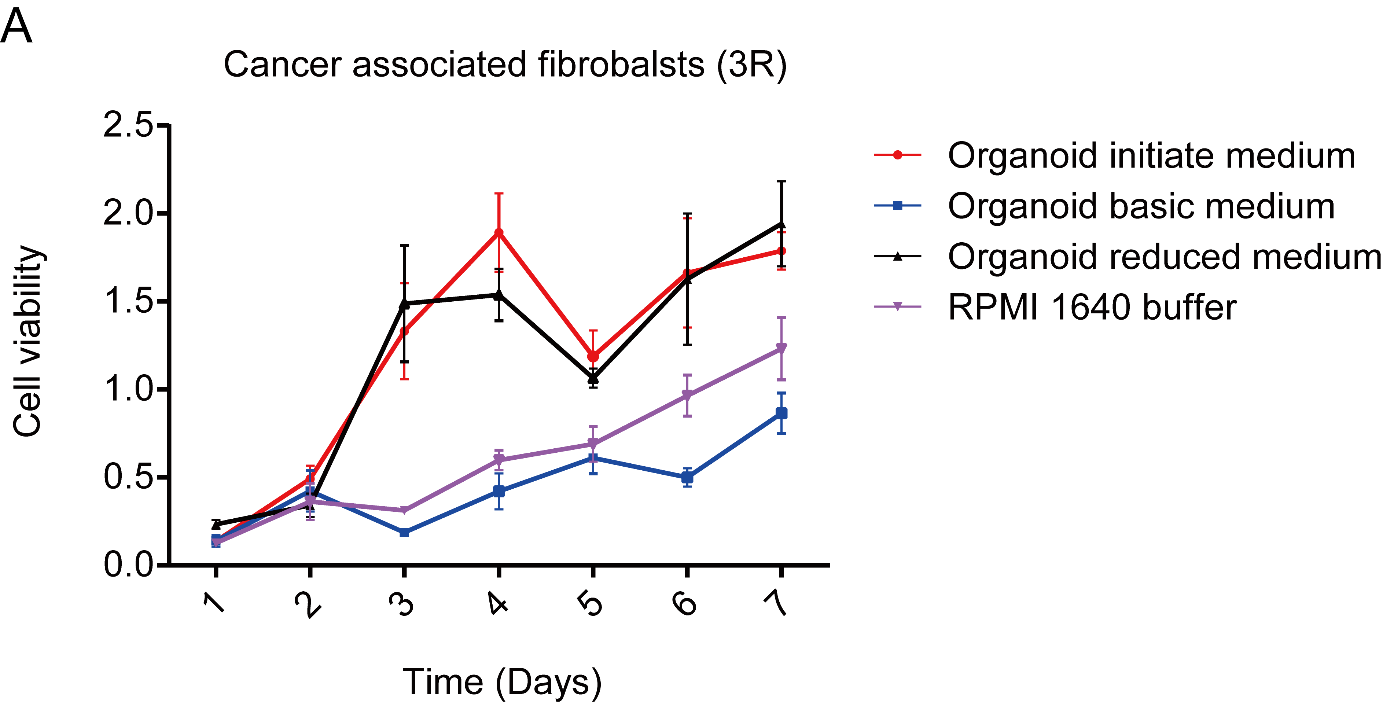
**

SFigure 2: CAFs were cultured using different organoid mediums and observed for activity.

**Supplementary Tables**

**Table 1，Four different organoid media components**

| Organoid initiate medium | Advanced DMEM/F12+1% penicillin/streptomycin+1% GlutaMAX+  10mmol/L HEPES, EGF (50ng/mL), FGF10 (100ng/mL), HGF  (50ng/mL), gastrin (10nmol/L), R-spondin 1(10% vol/vol), forskolin B27 (2%vol/vol), N2(1%, vol/vol), nicotiminade(10mmol/L), N-acetylcysteine (1.25μmol/L) |
| --- | --- |
| Organoid basic medium | Advanced DMEM/F12+1% penicillin/streptomycin+1% GlutaMAX  +10mmol/L HEPES |
| Organoid reduced medium | Advanced DMEM/F12+1% penicillin/streptomycin+1% GlutaMAX  +10mmol/L HEPES, EGF (50ng/mL), FGF10 (100 ng/mL), HGF (50ng/mL), gastrin (10nmol/L), R-spondin 1 (10% vol/vol), forskolin, N2 (1%, vol/vol), N-acetylcysteine (1.25μmol/L) |
| RMPI 1640 buffer | RMPI 1640+2%FCS |
